# Interleukin-27-dependent transcriptome signatures during neonatal sepsis

**DOI:** 10.3389/fimmu.2023.1124140

**Published:** 2023-02-20

**Authors:** Jessica M. Povroznik, Halima Akhter, Jordan K. Vance, Madhavi Annamanedi, Sebastian A. Dziadowicz, Lei Wang, Ashley M. Divens, Gangqing Hu, Cory M. Robinson

**Affiliations:** ^1^ Department of Microbiology, Immunology, & Cell Biology, West Virginia University School of Medicine, Morgantown, WV, United States; ^2^ Vaccine Development Center, West Virginia University Health Sciences Center, Morgantown, WV, United States

**Keywords:** interleukin-27, neonate, infection, sepsis, gram-negative, transcriptome, inflammation

## Abstract

Human newborns exhibit increased vulnerability and risk of mortality from infection that is consistent with key differences in the innate and adaptive immune responses relative to those in adult cells. We have previously shown an increase in the immune suppressive cytokine, IL-27, in neonatal cells and tissues from mice and humans. In a murine model of neonatal sepsis, mice deficient in IL-27 signaling exhibit reduced mortality, increased weight gain, and better control of bacteria with reduced systemic inflammation. To explore a reprogramming of the host response in the absence of IL-27 signaling, we profiled the transcriptome of the neonatal spleen during *Escherichia coli*-induced sepsis in wild-type (WT) and IL-27Rα-deficient (KO) mice. We identified 634 genes that were differentially expressed, and those most upregulated in WT mice were associated with inflammation, cytokine signaling, and G protein coupled receptor ligand binding and signaling. These genes failed to increase in the IL-27Rα KO mice. We further isolated an innate myeloid population enriched in macrophages from the spleens of control and infected WT neonates and observed similar changes in gene expression aligned with changes in chromatin accessibility. This supports macrophages as an innate myeloid population contributing to the inflammatory profile in septic WT pups. Collectively, our findings highlight the first report of improved pathogen clearance amidst a less inflammatory environment in IL-27Rα KO. This suggests a direct relationship between IL-27 signaling and bacterial killing. An improved response to infection that is not reliant upon heightened levels of inflammation offers new promise to the potential of antagonizing IL-27 as a host-directed therapy for neonates.

## Introduction

The neonatal period is a time in which newborns have increased vulnerability and the highest risk of death from infection. Increased susceptibility to infection in this period is generally considered reflective of differences in innate and adaptive immune function as compared with those of adult cells. Phenotypic and functional deficiencies have been reported in neonatal innate and adaptive immune cells that are not found in the adult immune system. For example, the neonatal immune system exhibits a Th2-biased/T cell regulatory response relative to adults (1), as well as an increased number of neutrophils that fail to successfully demonstrate chemotaxis or a response to chemotactic factors (2). Additionally, increased levels of T cells, B cells, NK cells, and monocytes are also found in neonates with distinct differences in their cell surface maker expression, antigen presentation, and control and elimination of bacteria compared to adult immune cells (3–5). An increase in the anti-inflammatory cytokine IL-27 is found in both neonatal murine serum, as well as macrophages derived from umbilical cord blood (6, 7). This finding is consistent with reports of decreased TNF-α, IFN-α, IFN-γ, IL-1β, IL-6, and IL-8 relative to adults (8, 9). Collectively, low levels of Th1 cytokines, high production of anti-inflammatory cytokines, and Th2-polarizing activity, all of which contribute to an increased susceptibility to infection of most pathogens, characterize the immune profile in neonates (1, 6, 7, 10, 11).

IL-27 is a heterodimeric cytokine of the IL-12 family that consists of the Epstein-Barr virus-induced gene 3 (EBI3) and IL-27p28 (also referred to as p28) proteins expressed from distinct genes (12). Similar to other members of the IL-12 family, IL-27 is a type-1 cytokine defined by its four-helix bundle and hematopoietin-receptor domain (13). Its receptor complex is dimeric and comprised of IL-27Rα (also referred to as WSX-1) and the glycoprotein gp130 (14). Activation of the IL-27 receptor complex results in an induction of JAK/STAT and p38 MAPK/ERK signaling cascades (15, 16). We have previously shown that IL-27 expression and protein secretion is elevated in macrophages obtained from human umbilical cord blood compared with those obtained from adult peripheral blood. We further demonstrated similar findings in mice in which total splenic transcript levels and IL-27 protein levels in macrophages are increased through approximately 3 weeks of life. In further agreement, serum IL-27 is increased throughout the neonatal period (6).

Human newborns exhibit increased risk of sepsis. The approximate rate of 1 case per 1,000 live births in the U.S. totaling greater than 75,000 per year increases significantly with low-birthweight (LBW) and pre-term infants (17, 18). These factors increase the mortality rate to nearly 25% (17). Beyond the emergent situation that sepsis represents in the neonate, the condition further predisposes to secondary infections and poor neurodevelopmental outcomes (17, 19). Recently, we established a murine neonatal model of *Escherichia coli* O1:K1:H7-induced sepsis to explore the impact of early life IL-27 levels on the host response to infection (20, 21). In this model, neonatal pups deficient in IL-27 signaling exhibit reduced mortality, increased weight gain, and better control of the bacterial burden with reduced systemic inflammation (20). Importantly, our model demonstrated a further increase in IL-27 expression during infection in the spleen, a known site of infection exhibiting high bacterial burden. Together, these results suggest that IL-27 opposes protective immunity and there is a reprogramming of the host response in the absence of IL-27 signaling. To further explore this possibility and potentially unlock a formula to promote increased control of bacteria in neonates, we profiled the neonatal transcriptome of the total spleen and splenic myeloid population in the presence or absence of infection in wild-type (WT) and IL-27 receptor (IL-27R)-α-deficient pups.

## Materials and methods

### Mice

Breeding pairs of C57BL/6 (WT) or IL-27Rα-deficient (KO) mice on a C57BL/6 genetic background were purchased from Jackson Laboratories (Bar Harbor, ME) and maintained under specific pathogen-free conditions in the vivarium at West Virginia University Health Sciences Center. Mice were maintained on a 12-h light/dark cycle and were fed/watered ad libitum. Male and female pups were used for experimental infection. Blood and tissues were collected from mice at the appropriate age by humane procedures.

### Bacteria

*Escherichia coli* strain O1:K1:H7 was obtained from the ATCC (Manassis, VA) and grown in Luria broth from a single colony isolated on Tryptic Soy agar (TSA). To prepare infectious inoculums, the bacteria were enumerated as described previously (21).

### Infection of neonatal mice

Neonatal pups (n=3 per group (control vs. infected) per genotype (WT *vs*. KO)) at 4 days were inoculated subcutaneously in the scapular region with *E. coli* O1:K1:H7 using a 28-gauge insulin needle as described previously (20, 21). The bacteria were washed with PBS, centrifuged at 2,000 x g for 5 min, and suspended in a volume of PBS equivalent to an inoculum of 50 μL/mouse. The target inoculum was 10^6^ CFUs per mouse and actual inoculums as determined by standard plate counts, ranged from 4.8 x 10^5^ to 1.6 x 10^6^ CFUs per mouse. Vehicle (PBS)-inoculated pups were identified from *E. coli*-infected pups using a tail snip. The weights of mice were recorded immediately prior to infection, and at 24 h immediately prior to euthanasia. At 24 h post-infection, mice were harvested and individual mice (*E. coli* infected and tail-snipped PBS pups) were numbered; all downstream experiments were associated with the same pre-identified mice. Spleens isolated from pups were placed in PBS on ice. Blood was deposited in tubes that contained 5 μL of 500 mM ethylenediamine tetraacetate acid (EDTA) and placed on ice. The bacterial burden in the blood was enumerated by serial dilution and standard plating on TSA.

### Enrichment for myeloid cells

Four-day old C57BL/6 (n=10) and IL-27Rα-deficient (n=11) pups infected for 24 h were humanely euthanized and the spleens harvested as described above. To obtain sufficient cells for downstream analysis, spleens were pooled based on infection status (control *vs*. infected) to generate three control and three infected samples for sequencing from each genotype. Single cell suspensions were generated by crushing the spleens through a 40 µM strainer. Cells were placed in complete Dulbecco’s Modified eagle Medium (DMEM) with 30% fetal bovine serum (FBS). The myeloid-enriched fraction of mononuclear cells was isolated using Optiprep™ (Sigma) density gradient centrifugation as described previously and scaled down four-fold to accommodate the smaller number of cells in neonatal spleens (22). The resulting myeloid cells were washed, suspended in complete DMEM with 10% FBS, and counted (1-5x10^5^ cells per sample) before immediately proceeding to processing for ATAC and RNA sequencing.

### RNA isolation

Spleens were homogenized in TRI Reagent^®^ (Molecular Research Center, Cincinnati, OH). Using the commercial product protocol, the upper aqueous layer following phase separation was mixed with an equal volume of 75% ethanol and transferred to E.Z.N.A.^®^ RNA isolation columns (Omega Biotek, Norcross, GA). The manufacturer’s instructions were followed to complete tissue RNA isolation. RNA was isolated from approximately 7.5x10^4^ splenic myeloid cells using the RNeasy^®^ Plus Mini Kit (Qiagen, Hilden, Germany, Cat# 74134) according to the manufacturer’s instructions. RNA from cells or tissue was quantified using a Qubit Fluorometer and the RNA integrity was analyzed using an Agilent Bioanalyzer 2100 (Agilent technologies, Santa Clara, CA).

### Quantitative real time PCR

First-strand cDNA was synthesized using the iScript™ cDNA synthesis kit (Bio-Rad, Hercules, CA). Quantitative PCR reactions included cDNA diluted four-fold, gene-specific TaqMan^®^ primer probe sets (Applied Biosystems^®^, Foster City, CA), and iQ™ Supermix (Bio-Rad, Hercules, CA). Cycling was performed in triplicate using a StepOnePlus™ Real-Time detection system (Applied Biosystems^®^, Foster City, CA). Gene-specific amplification was normalized to GAPDH as an internal reference gene and expressed as log_2_ relative gene expression compared to control spleen using the formula 2^-ΔΔCt^.

### RNA sequencing

Library preparation and sequencing were performed at the Genomics Core Facility of West Virginia University and Marshall University, respectively. RNA-Seq libraries were built using KAPA mRNA HyperPrep Kit (KAPA Biosystems, Wilmington MA) with 100-500 ng of total RNA according to manufacturer’s recommendation. The libraries were quantified using a Qubit Fluorometer, and the quality determined by Bioanalyzer High Sensitivity DNA Analysis (Agilent, Santa Clara, CA). Libraries were sequenced at the Genomics Core Facility at Marshall University with an Illumina NextSeq2000 (Illumina, San Diego, CA).

### Assay for transposase-accessible chromatin followed by sequencing

ATAC libraries were prepared from approximately 50 to 75 thousand of mononuclear cells using a Tagment DNA Enzyme Kit from Illumina (Cat # 20034198) by following the Omni-ATAC protocol (23). The ATAC libraries were run on 2% agarose gel and gel band corresponding to ~200–600 bp was cut to elute DNA fragments using the MinElute^®^ Gel Extraction Kit (Qiagen), subjected to sequencing at the Genomics Core Facility at Marshall University with Illumina Nextseq2000.

### Bioinformatic data analysis

RNA-Seq data analysis followed our previous work (24). Pair-end RNA-Seq read were mapped to the mouse genome (mm10) by subread v2.0.1 with default parameters (25). The number of RNA-Seq fragments mapped to transcripts was summarized at gene level by featureCounts (26). Gene transcription level was quantified by RPKM (27). Differentially expressed genes were predicted by EdgeR3 (FDR < 0.05 and fold change > 2) (28). In this analysis, we required genes to be expressed at least in one of the compared conditions (RPKM>3 and log2 (count per million)>0). Meanwhile, we excluded genes exhibiting a substantial variation (maximal/minimal > 2) in expression across samples from the same condition. Gene Set Enrichment Analysis against Reactome pathways, Gene Ontology (biological processes), and Hallmark gene sets from MSigDB (29) were done by GSEA (30). Gene ontology enrichment analysis on biological processes (BP3) was done with the online DAVID bioinformatics resource (31), where non-redundant hits were visualized as bubble plots by REVIGO (32).

For ATAC-seq data analysis, sequencing reads was aligned to mouse genome (mm10) by Bowtie2 (33). Visualization of reads density across selected gene loci was done by IGV (34). ATAC-seq read enriched regions were identified by MACS3 (q-value < 0.001) (35). Differentially accessible regions (DARs) were predicted by EdgeR3 (FDR < 0.05 and fold change > 1.5) (28). Target genes of DARs were predicted by the online tool GREAT (36).

Integrative analysis comparing *E. coli*-induced expression changes between WT spleen and macrophages isolated from the spleen (Compromised transcriptome response to *E. coli* in macrophages in the absence of IL-27Ra section) was done with GSEA. In this analysis, we focused on genes identified as up-regulated or down-regulated by *E. coli* infection in WT macrophages. The GSEA evaluates their overall ranks on a red-to-blue spectrum which represents *E. coli*-induced expression changes in the spleen (red: higher expression after infection; blue: lower expression after infection). An overall rank to the left side of the spectrum indicates an upregulation in the spleen, while an overall rank to the right side indicated an overall down-regulation.

### Statistical analysis

Weight, bacterial burden, and gene expression data (Mean ± SEM) were analyzed with two-way ANOVAs for each given dependent measure using Prism 8 (GraphPad, San Diego, CA). The threshold for statistical significance was set at alpha = 0.05.

## Results

### IL-27 signaling compromises control of bacteria during neonatal sepsis

**Figure 1:**
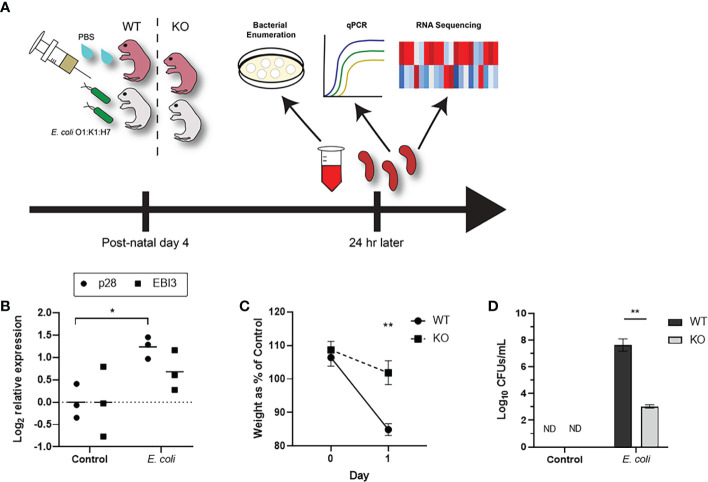
IL-27 signaling compromises control of bacteria during neonatal sepsis. **(A)** A schematic to illustrate the study design to pair transcriptional analysis with infection related outcomes. Neonatal mice were subcutaneously inoculated with *E. coli* O1:K1:H7 (n=3) or PBS (n=3) as a control on day 4 of life. **(B)** The expression of IL-27p28 or EBI3 in the infected spleen at 1-day post-infection was determined relative to tissues from uninfected controls by real-time PCR using the formula 2^−ΔΔCt^. Each symbol represents an individual animal analyzed further in this study with the mean for each group displayed. **(C)** To compare WT and IL-27Rα^-/-^ (KO) weights directly, the percent change for the infected pups (n=3) relative to the control pups (n=3) was represented for each day. A two-way ANOVA and Bonferroni multiple-comparison test were used to determine statistical significance between WT and KO groups. **, P =0.0041. **(D)** The same mice used in **(B, C)** were used to determine bacterial burdens in the blood for WT and KO infected neonates at 1-day post-infection. Data is shown as the mean log_10_ transformed CFUs/mL ± SEM. An unpaired *t* test was used to determine statistical significance between WT and KO infected neonates.An unpaired samples t test was used to determine statistical significance between control and infected groups. *, P = 0.0134 **, P = 0.0067.

Neonates exhibit elevated levels of IL-27 in the spleen and blood at resting state relative to adults, and these levels continue to rise during infection peaking at 24 h (7, 20). IL-27Rα^-/-^ (KO) mice have an allelic exchange of a neomycin resistance cassette for exon 2 in the C57BL/6 background and fail to produce a functional receptor protein (**Supplementary Figure 1**). These mice exhibit superior control of bacteria and improved outcomes during infection (20). To understand how the transcriptome may be influenced by IL-27 and further identify a transcriptional signature associated with improved host response, we infected WT or IL-27Rα^-/-^ neonatal mice at day 4 of life as described previously (**Figure 1A**). These mice were euthanized at day 1 post-infection, a time that corresponds with peak IL-27 levels and a critical period for control of bacteria (20). Phenotypic changes are observed in our neonatal sepsis model that are consistent with an increase in IL-27 gene expression during systemic infection (**Figure 1B**). Expression of the IL-27p28 subunit is considered rate limiting for assembly of the bioactive cytokine; EBI3 expression is more constitutive and also not exclusive to IL-27. IL-27Rα^-/-^ (KO) neonates that fail to respond to IL-27 exhibit improved maintenance of weight compared to WT neonates (**Figure 1C**). Relative to KO pups, WT infected pups exhibited a 16.96% decrease in weight compared to their uninfected WT littermates 1 day-post-infection; this decrease in weight between infected and uninfected KO pups was not observed (**Figure 1C**). This is further consistent with differences in pathogen burden (**Figure 1D**). Infected WT pups demonstrated a profound increase in bacterial burdens in the blood 1-day post-infection. Infected KO pups exhibited significantly fewer CFUs in the blood than infected WT pups (**Figure 1C**).

### The transcriptional response to *E. coli* infection in the spleen

**Figure 2:**
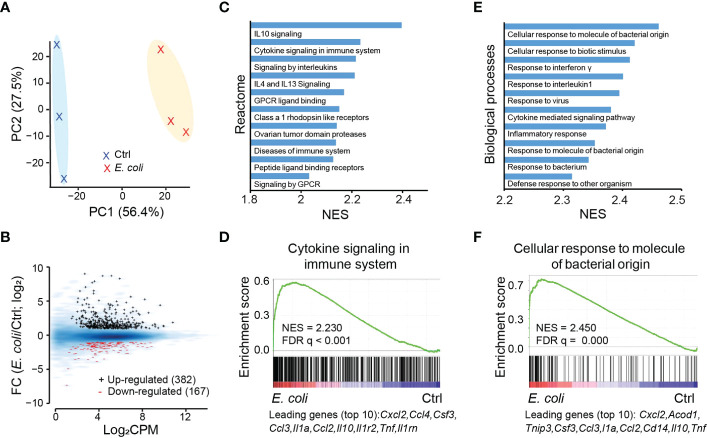
Transcriptional response to *E. coli* infection in the spleen. **(A)** PCA analysis based on gene expression showing a clear separation of *E. coli* treated samples from the untreated samples (Ctrl). **(B)** MA plot for genes differentially expressed between *E. coli* treated and Ctrl samples. Black +: genes upregulated after *E. coli* infection; red -: genes downregulated after *E. coli* infection. Blue smoothed heat map: all expressed genes. **(C)** Top 10 gene sets for Reactome pathways that are upregulated in the samples treated with *E. coli* as predicted from GSEA analysis (FDR q < 0.01). NES: normalized enrichment score. **(D)** GSEA analysis for expressed genes ranked by fold change of expression (*E. coli*/Ctrl) against the Reactome gene set “cytokine signaling in immune system”, with top 10 up-regulated genes indicated at the bottom. **(E)** Top 10 gene sets for gene ontology “biological processes” that are upregulated in samples treated with *E. coli* as predicted from GSEA (FDR q < 0.01 for all). **(F)** GSEA analysis for expressed genes ranked by fold change of expression *E. coli*/Ctrl) against the biological process “cellular response to molecule of bacterial origin”, with top 10 up-regulated genes indicated at the bottom.

To understand the impact of IL-27 on regulation of the neonatal host response to infection, we profiled the transcriptional response in uninfected control and *E. coli* O1:K1:H7-infected neonates at 1-day post-infection. This period is consistent with peak serum IL-27 levels in our model of neonatal sepsis (20). The expression profiles in infected pups show clear separation according to principal component analysis (PCA) (**Figure 2A**). Although the magnitude and abundance of genes that are differentially increased in expression during infection is greater, there is also a clear set of genes that are differentially decreased in expression (**Figure 2B**; **Supplementary Table 1**). According to Gene Set Enrichment Analysis (GSEA) against Reactome (37), the most up-regulated gene set pathways are associated with inflammation, cytokine signaling, and G protein coupled receptor ligand binding and signaling (**Figure 2C**). The results are not unexpected as an intense inflammatory response ensues during neonatal sepsis that frequently manifests as a “cytokine storm” and a potentially pathological level of inflammation. The top ten individual genes with the greatest magnitude of increased expression in one top gene set “cytokine signaling in immune system” are all cytokines and chemokines (**Figure 2D**). Among these *cxcl2*, *ccl4*, *ccl3*, and *ccl2* are all chemotactic factors involved with recruitment of neutrophils and monocytes to sites of inflammation. Although speculative, it seems likely that IL-10, an anti-inflammatory factory, is increased in expression as a product of a robust inflammatory response and part of a negative feedback inflammatory loop (**Figure 2D**). Similarly, the top up-regulated biological processes identified by gene ontology analysis aligned with response to bacterial and viral infection, cytokine signaling, and inflammatory response (**Figure 2E**). Many of the top ten individual genes (i.e. *cxcl2*, *csf3*, *ccl3*, *il1a*, *ccl2, il10*, and *tnf*) with the greatest magnitude of increased regulation in expression in the biological process “cellular response to molecular of bacteria origin” (**Figure 2F**) overlap with leading genes from GSEA analysis on “cytokine signaling in immune system” (**Figure 2D**). Similar to IL-10 above, cis-aconitate decarboxylase (*acod1*) is a negative regulator (38, 39) of inflammatory responses and may be upregulated to counterbalance the robust inflammatory response to bacteria (**Figure 2F**). However, *acod1* has also been shown to contribute to antimicrobial activity of macrophages by catalyzing itaconic acid production (39). TNFAIP3-interacting protein 3 (TNIP3) also inhibits NF-κB-dependent gene expression in response to LPS (40), most likely a host attempt to regulate prolonged inflammatory signaling.

REACTOME pathways were less intuitive in IL-27 biology (**Supplementary Figures 2A, B**). Elastic fibers are scaffolded and crosslinked to form an insoluble polymer that provides tissue elasticity and resistance to mechanical strain (41). Defects in elastic fiber formation result in a variety of tissue pathologies (41). This is the first report of an influence of IL-27 on this gene set pathway. Genes with functional roles in transcriptional regulation by small RNAs may have implications in host control of the inflammatory response, a consistent theme in the infected transcriptome (**Supplementary Figure 2A**). The expression of these genes may be negatively regulated by the persistent and increasing bacterial burden. Biological processes identified by gene ontology enrichment analysis downregulated during infection show significant enrichment in genes that align with DNA replication and cell cycle pathways (**Supplementary Figures 2C, D**). Although speculative, cellular damage amidst a strong inflammatory response and high bacterial burden may oppose cellular replication pathways.

A prior report by Li, et al. (42) explored changes to the liver transcriptome during *E. coli*-induced adult murine sepsis. Genes that were differentially expressed during infection compared to control included sets that were both increased and decreased (**Supplementary Figure 3A**). Not unexpectedly, biological processes associated with innate immune function and host response to infection were up-regulated through GSEA (**Supplementary Figure 3B**). Down-regulated biological processes were enriched for those involved in central metabolism (**Supplementary Figure 3C**). This finding is also not surprising given the role of the liver in energy metabolism. Further analysis revealed that 222 up-regulated genes and 35 down-regulated genes in liver exhibited consistent expression changes in our study in spleen (**Supplementary Figure 3D**). The shared up-regulated genes reveal a common emphasis in biological processes associated with innate and adaptive immune response, wound healing, cytokine production, cellular adhesion, and signal transduction to name a few (**Supplementary Figure 3E**).

### Transcriptional changes induced by IL-27Rα depletion before *E. coli* infection

**Figure 3:**
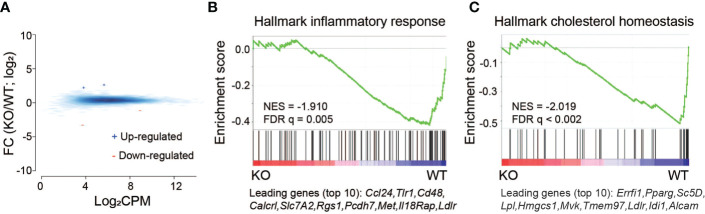
Basal level transcriptional changes induced by IL-27Rα depletion without *E. coli* infection. **(A)** MA plot for genes differentially expressed in spleen between IL-27Rα depletion (KO) and wild-type control (WT). Blue +: genes upregulated by IL27Rα KO; red -: genes down-regulated by IL27Rα KO. Blue smoothed heat map: all expressed genes. **(B)** GSEA analysis for expressed genes ranked by fold change of expression (KO/WT) against the MSigDB Hallmark gene set “inflammatory response”, with top 10 down-regulated genes shown at the bottom. **(C)** GSEA analysis for expressed genes ranked by fold change of expression (KO/WT) against the MSigDB Hallmark gene set “cholesterol homeostasis”, with top 10 down-regulated genes shown at the bottom.

IL-27Rα^-/-^ mice do not express a functional receptor and cannot respond to IL-27. The change in global gene expression with IL27Rα depletion compared to WT in the absence of infection is not dramatic and reveals only a limited number of differentially expressed genes (**Figure 3A**; **Supplementary Table 1**). Of importance at the gene set level, GSEA analysis revealed a subset of genes involved in inflammatory response and cholesterol homeostasis are preferentially downregulated in the absence of the IL27Rα (**Figures 3B, C**). Overall, it is not clear at this time what the connection may be with the downregulated inflammatory response genes and outcomes reported during infection, but some may be part of a reprogramming of the host response to be less reactive. Regulation of cholesterol homeostasis by IL-27 may have important metabolic implications that are relevant to neonatal physiology. That expression of these genes was lower in IL-27Rα-infected pups may suggest a link with other metabolic pathways and improved maintenance of weight observed during infection. The low-density lipoprotein receptor (*ldlr*, **Figure 3C**) that can potentiate inflammatory responses (43) and is increased in expression in WT pups, is also interrelated with cholesterol homeostasis (44). These data describing transcriptional regulation by IL-27 signaling in the absence of infection are novel findings.

### Transcriptional reprogramming in response to *E. coli* requires IL-27Rα

**Figure 4:**
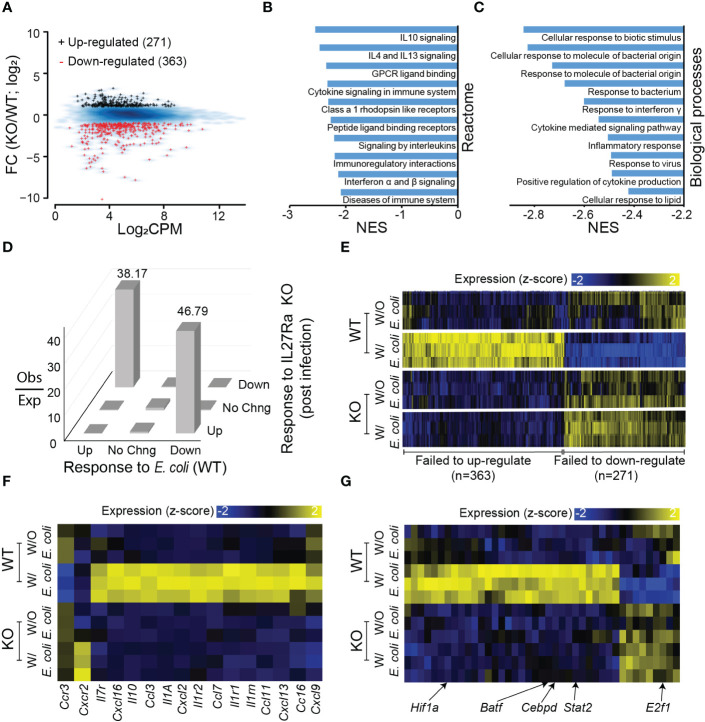
Transcriptional reprogramming in response to *E. coli* requires IL-27Rα. **(A)** MA plot for genes differentially expressed in the spleen between IL-27Rα-depleted (KO) and WT control following *E. coli* infection. Black +: genes upregulated by IL-27Rα KO; red +: genes down-regulated by IL27Rα KO. Blue smooth heat map: all expressed genes. **(B)** Top 10 gene sets for Reactome pathways that are upregulated post *E. coli* infection in the KO samples as predicted from GSEA (FDR q < 0.01). NES: normalized enrichment score. **(C)** Top 10 gene sets for gene ontologies in biological processes that are upregulated post *E. coli* infection in KO samples as predicted from GSEA (FDR q < 0.01). **(D)** Observed versus expected number of genes (z-axis), sorted based on expression changes induced by *E. coli* infection in control samples where IL-27Rα is intact (x-axis) and expression changes induced by IL-27Rα depletion in samples post *E. coli* infection (y-axis). Abbreviations on x-axis: Up = genes up-regulated by *E. coli* infection as compared to no-infection, both in WT cells; Down = genes down-regulated; No Chng = genes with no significant expression changes. Abbreviations on y-axis: Up = genes up-regulated by IL27Ra KO as compared to WT, both in cells post *E. coli* infection; Down = genes down-regulated; No Chng = genes with no significant expression changes. Abbreviations on z-axis: Obs = Observed number of genes; Exp = expected gene number of genes. **(E)** Heatmap visualization of expression values for genes that are differentially expressed between WT and KO samples in the presence or absence of *E. coli* infection. Each mouse is represented by a separate row. Genes are hierarchically clustered and manually annotated into two groups: failed to up-regulate or failed to down-regulate in gene expression during *E. coli* infection in the absence of IL-27Rα. The expression values for each gene (column) are z-score transformed across samples (also applied to panes **F, G**). **(F)** Similar to panel E but for a subset of genes annotated as chemokine receptors, interleukins, and chemokine ligands. **(G)** Similar to panel **(E)** but for a subset of genes annotated as transcription factors with representatives indicated.

Since the number of IL-27Rα-dependent genes was marginal at base level and we observed phenotypic differences in neonates during infection, we further explored the transcriptional response during systemic *E. coli* infection. There were 634 genes that were differentially expressed between WT and KO neonates during infection; 271 were significantly upregulated and 363 were downregulated with IL-27Rα deficiency (**Figure 4A**; **Supplementary Table 1**). According to GSEA, the most preferentially downregulated pathways and biological processes in the KO pups are associated with cytokine and chemokine signaling (**Figure 4B**) and response to cytokines or bacteria (**Figure 4C**). Collectively, this highlights a less inflammatory environment that is not expected given the anti-inflammatory/suppressive activity of IL-27. However, it is consistent with a lower bacterial burden (**Figure 1D**). This suggests that improved bacterial clearance in the absence of IL-27 signaling does not require a heightened inflammatory state, and instead, there is a direct relationship between IL-27 signaling and bacterial killing. The most preferentially upregulated pathways and biological processes in the KO pups are less intuitively involved in host response to infection with a central theme of DNA replication, maintenance, and repair (**Supplementary Figures 4A, B**). This may be reflective of overall improved physiology in KO pups that is disrupted in WT animals.

Further analysis indicated that genes upregulated by *E. coli* infection were generally downregulated post-infection in the absence of IL-27Rα with a similar observation made for genes downregulated by *E. coli* infection (**Figure 4D**). We confirmed the observations by visually inspecting gene expression changes prior to and after infection with *E. coli* in the WT and KO; 363 genes failed to increase, and 271 genes failed to decrease in the KO group following infection (**Figure 4E**). Among the genes that fail to increase during infection in the absence of IL-27 signaling are chemokines, chemokine receptors, and interleukins (**Figure 4F**; **Supplementary Figure 4C**). This further supports a less inflammatory environment in the infected KO compared to WT neonates. Our data further suggests that this is the product of a transcriptional reprogramming that occurs in WT but not KO neonates during infection. Expression of genes for notable transcriptional activators such as Stat-2, Batf, XBP1, and CEBP-δ that regulate host inflammatory responses are not increased in the spleen of IL-27Rα-deficient pups (**Figure 4G**). Among TFs that failed to down-regulated their expression levels (**Figure 4G**), E2f1 is well-characterized for its functions in regulating cell cycle (45). Collectively these data demonstrate an improved response to infection that is not reliant upon heightened levels of inflammation as have been shown with other mouse models of chronic tuberculosis (46, 47). This offers new promise to the potential of antagonizing IL-27 as a host-directed therapy for neonates without concern for driving inflammatory responses that are pathological to host tissues.

### Compromised transcriptome response to *E. coli* in macrophages in the absence of IL-27Rα

**Figure 5:**
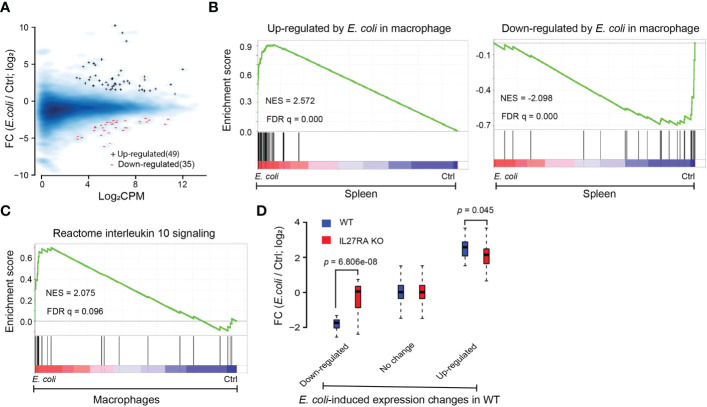
Compromised transcriptional response to *E.* coli in macrophage-enriched cells deficient in IL-27Rα. **(A)** MA plot for genes differentially expressed between *E. coli*-treated and non-treated (Ctrl) WT macrophages. Blue +: genes upregulated after *E. coli* infection; red -: genes down-regulated after *E. coli* infection; Blue smooth heat map: all expressed genes; FC, Fold change; CPM, Counts per million reads. **(B)** GSEA analysis for expressed genes ranked by fold change of expression (*E. coli*/Ctrl in WT spleen) from high to low on the red-to-blue spectrum against genes upregulated (vertical bars on the left panel) or downregulated (vertical bars on the right panel) by *E. coli* in WT macrophages. NES: normalized enrichment score. **(C)** GSEA analysis for expressed genes ranked by the fold change of expression (*E. coli*/Ctrl in WT macrophages) from high to low on the red-to-blue spectrum against genes from Reactome IL10 signaling pathway (vertical bars). **(D)** Boxplots of expression changes induced by *E. coli* in macrophages deficient in IL-27Rα (KO; red boxplots) and in WT macrophages (blue boxplots) for genes sorted based on their expression response to *E. coli* in WT macrophages. Down-regulated: genes down-regulated by *E. coli* as compared to nontreatment (Ctrl) in WT macrophages; up-regulated: genes up-regulated; No change: genes with no significant expression changes; *P*-value by paired *t*-test. FC, Fold change.

Improved control of bacteria was observed in infected KO pups, and we have previously demonstrated that IL-27 impairs macrophage control of a variety of bacteria, including *E. coli* (7, 20, 48–51). We therefore isolated the myeloid fraction of cells enriched with macrophages from the spleens of WT and IL-27Rα KO neonatal pups challenged with *E. coli*. Gene expression data analysis with RNA sequencing identified dozens of differentially expressed genes in response to *E. coli* infection in WT mice (**Figure 5A**; **Supplementary Table 1**). We aligned the differentially expressed genes defined from the macrophage population to the *E. coli*-induced expression changes at the spleen tissue level using GSEA and observed consistent changes in expression (**Figure 5B**). As expected, *E. coli* infection upregulated IL-10 signaling in macrophages, though the level of significance was as modest as FDR q = 0.096 (**Figure 5C**). Interestingly, compared to WT mice, the expression changes induced by *E. coli* infection were generally smaller in the KO mice (**Figure 5D**), suggesting that in the absence of IL-27Rα the transcriptional program of macrophages became less responsive to *E. coli* infection.

To understand potential epigenetic regulatory mechanisms, we extended the analysis to profile chromatin accessibility in the same sets of macrophage-enriched cells for which RNA sequencing was performed. The analysis identified genes associated with multiple genomic regions that showed an increase or decrease in chromatin accessibility in response to *E. coli*. Examples include *s100a9* and *mrc1* (**Supplementary Figure 5A**). Proteins in the S100 family are among the most abundant cytoplasmic proteins in myeloid-differentiated cells that are released during activation of phagocytes and known to play a significant role in the pathogenesis of sepsis (52–54), while MRC1 is a C-type lectin receptor and expression is generally considered a marker of a more M2-like or regulatory macrophage polarization (55). As expected, genes associated with regulatory regions that increased in accessibility were up-regulated in expression level (**Supplementary Figure 5B**), while those associated with decrease in accessibility were down-regulated (**Supplementary Figure 5B**). Consistent with expression changes, in the absence of IL-27Rα, *E. coli*-induced changes in chromatin accessibility were generally smaller than in the WT mice (**Supplementary Figure 5C**).

## Discussion

Neonatal pups deficient in IL-27 signaling exhibit a striking improvement in survival and reduced signs of morbidity that is highlighted by improved control of bacteria and reduced systemic inflammation during gram-negative bacterial sepsis (20). This suggests a transcriptional reprogramming to be unlocked in this genotype for a superior host response. Since the spleen is a known site for IL-27 expression during neonatal sepsis that correlates with a high bacterial burden in WT neonates, we profiled the transcriptome of this tissue. The finding that gene set pathways increased during infection of WT neonatal mice were enriched with those associated with inflammation, cytokine signaling, and G protein coupled receptor ligand binding and signaling is not surprising. As the presence of bacteria and bacterial-derived products drive inflammation, expression of inflammatory markers and host defense pathways are expected to increase. Massive inflammation is a hallmark of bacterial sepsis. That genes in these same pathways are not increased in IL-27Rα KO neonatal pups during infection is the most compelling finding in this report. This can be explained by the influence of IL-27 on control of bacteria. With improved control of bacteria in IL-27Rα KO neonates, there is a reduced concentration of inflammatory stimuli. However, this comes in contrast to other reports where microbial burdens are reduced, but at the consequence of excessive inflammation.

IL-27 is a blessing and a curse in chronic infections. IL-27 can balance inflammation but promote pathogen survival. In the absence of IL-27 protein or signaling, numerous reports demonstrate that pathogen burdens are more controlled, but inflammation is elevated and consistent with enhanced pathology. The absence of IL-27-mediated signaling promotes a better control of mycobacterial growth during experimental tuberculosis but also leads to a chronic hyperinflammation and immunopathology later during infection (47). IL-27Rα-deficient mice infected with *Toxoplasma gondii* control parasite replication but are unable to downregulate T cell-mediated inflammation and exhibit excessive production of IFN-γ, persistence of highly activated T cells, and enhanced T cell proliferation (56). Similar findings are observed in experimental mouse models of infection by *Trypanosoma cruzi* (57), *Leishmania donovani* (58, 59), and the parasitic helminth *Trichuris muris* (58). Even in acute adult septic peritonitis, reduced bacterial clearance in the absence of IL-27 signaling was associated with a neutrophilic recruitment and inflammatory burst (60). In contrast to prior work, here we report a comprehensive benefit to the absence of IL-27 signaling. Most notably, improved control of bacteria is not associated with excessive inflammation. In contrast, inflammation is controlled and consistent with improved health. This highlights an influence of IL-27 on mechanisms that operate in phagocytic cells to kill bacteria. IL-27 has been directly implicated in lysosomal activity (50, 51). Vacuolar-ATPase expression is limited by IL-27 treatment with the consequence of impaired lysosomal acidification, and reducing killing of *Pseudomonas aeruginosa*, *Staphylococcus aureus*, and mycobacteria (50, 51). Consistent with these observations, macrophages and a mixed population of Ly6B.2^+^ phagocytes from IL-27Rα-deficient mice killed K1-encapsulated *E. coli* at an enhanced rate compared to WT counterparts (20). It is notable that in this report, IL-10 was among the genes that failed to increase during infection in the spleen of IL-27Rα^-/-^ pups. This further supports the idea that reduced inflammation in the absence of IL-27 signaling is a product of enhanced bacterial killing and not the result of increased expression of compensatory immune suppressive factors.

The significance of an overall lower expression of the inflammatory response genes at baseline in the absence of IL-27 signaling remains to be determined. However, many are consistent with priming the immune responsive to be less reactive. A leading gene, chemokine CCL24, is chemotactic for eosinophils, resting T cells, and neutrophils (61). CD48 interacts with bacterial fimbriae and could prime phagocytic cells for better recognition and clearance of bacteria (62). CD48 also is involved in cell adhesion, co-stimulation of lymphocytes, and activation of antigen presenting cells (63). TLR1 cooperates with TLR2 to sense triacylated lipoproteins from bacteria (64, 65). Regulator of G-protein signaling 1 (RGS1) regulates inflammation in macrophages; silencing of RGS1 limited NF-κB signaling and inflammatory cytokine production (66). IL18RAP is an accessory subunit of the receptor for interleukin-18 and mediates cytokine signaling important in mounting cell-mediated immune responses (67).

Among other unexpected findings in this report were genes enriched for cholesterol homeostasis in spleens from WT pups that were modestly down-regulated in IL-27Rα-deficient neonates. Although speculative at this time, this finding could provide a metabolic advantage linked with an ability to thrive and maintain body weight, wherein an increase in gene pathways associated with cholesterol homeostasis in non-infected WT pups could prime the pups for lipid dysregulation during an infected state. As such, it is already established that pediatric sepsis patients exhibit an altered lipid profile. Clinical findings indicate weight loss, as well as a negative correlation between C-reactive protein, a common marker of inflammation, and triglycerides, high-density lipoproteins, low-density lipoproteins, and apolipoprotein levels (68).

Congruent to changes in the spleen during infection, we also demonstrated changes in macrophages isolated from the myeloid fraction of the spleens in WT and KO pups. These changes in gene expression demonstrated through chromatin accessibility modulation align with changes in the splenic tissue transcriptome, wherein infected KO pups demonstrated a compromised change in chromatin accessibility relative to WT pups. These results are not surprising given the role of macrophages as dominant IL-27 producers that can also respond to the cytokine (6, 7, 49, 50). One consequence of IL-27 signaling is decreased lysosomal activity; macrophages exhibit improved lysosomal acidification, proteolytic activity, and bacterial clearance when IL-27 is neutralized (51). Merging those findings with those reported here, IL-27 opposes clearance of bacteria that promotes continued and elevated inflammation.

In summary, we present novel findings that suggest a transcriptional reprogramming of the host response in splenic tissue in the absence of IL-27 signaling during *E.coli*-induced infection in a neonatal murine sepsis model. Of note, neonatal pups deficient in IL-27Rα exhibited decreased mortality, improved weight gain, reduced inflammation, and enhanced control of bacterial growth during infection when compared to their WT counterparts. These physiologic data were correlated to bioinformatic analyses, which revealed an up-regulation in gene set pathways primarily associated with inflammation, cytokine signaling, and G protein coupled receptor signaling in WT pups during infection; these same gene set pathways failed to increase in IL-27Rα-deficient pups. We put forth that reduction of inflammation in IL-27Rα-deficient pups is associated with increased control of bacteria, and that, manipulation of IL-27 signaling in a clinical setting could improve overall outcome through a combined mechanism that balances reduced pathogen survival with dampening excessive pathology-inducing inflammation.

## Data availability statement

The datasets presented in this study can be found in online repositories. The names of the repository/repositories and accession number(s) can be found below: GSE220050 (GEO).

## Ethics statement

The animal study was reviewed and approved by West Virginia University Institutional Animal Care and Use Committee.

## Author contributions

JP, MA, JV, SD, AD, and CR performed the investigations. HA, LW, and GH performed the bioinformatics analysis. JP, HA, JV, GH, and CR wrote the original draft and reviewed the manuscript. CR conceived the study. All authors contributed to the article and approved the submitted version.

## Supplementary material

Supplementary Table 1 List of differentially expressed genes for **Figures 2B**, **3A**, **4A**, and **5A**.

## References

[1] Adkins B Leclerc C Marshall-Clarke S. Neonatal adaptive immunity comes of age. Nat Rev Immunol (2004) 4(7):553–64. doi: 10.1038/nri1394 15229474

[2] Weinberger B Laskin DL Mariano TM Sunil VR DeCoste CJ Heck DE. Mechanisms underlying reduced responsiveness of neonatal neutrophils to distinct chemoattractants. J Leukoc Biol (2001) 70(6):969–76. doi: 10.1189/jlb.70.6.969 PMC4027972 11739560

[3] Kim SK Keeney SE Alpard SK Schmalstieg FC. Comparison of l-selectin and Cd11b on neutrophils of adults and neonates during the first month of life. Pediatr Res (2003) 53(1):132–6. doi: 10.1203/00006450-200301000-00022 12508092

[4] Le Garff-Tavernier M Béziat V Decocq J Siguret V Gandjbakhch F Pautas E. Human nk cells display major phenotypic and functional changes over the life span. Aging Cell (2010) 9(4):527–35. doi: 10.1111/j.1474-9726.2010.00584.x 20477761

[5] Velilla PA Rugeles MT Chougnet CA. Defective antigen-presenting cell function in human neonates. Clin Immunol (2006) 121(3):251–9. doi: 10.1016/j.clim.2006.08.010 PMC1764492 17010668

[6] Gleave Parson M Grimmett J Vance JK Witt MR Seman BG Rawson TW. Murine myeloid-derived suppressor cells are a source of elevated levels of interleukin-27 in early life and compromise control of bacterial infection. Immunol Cell Biol (2019) 97(5):445–56. doi: 10.1111/imcb.12224 PMC6536317 30575117

[7] Kraft JD Horzempa J Davis C Jung JY Peña MM Robinson CM. Neonatal macrophages express elevated levels of interleukin-27 that oppose immune responses. Immunology (2013) 139(4):484–93. doi: 10.1111/imm.12095 PMC3719065 23464355

[8] Kollmann TR Crabtree J Rein-Weston A Blimkie D Thommai F Wang XY. Neonatal innate tlr-mediated responses are distinct from those of adults. J Immunol (2009) 183(11):7150–60. doi: 10.4049/jimmunol.0901481 PMC4556237 19917677

[9] Schultz C Temming P Bucsky P Göpel W Strunk T Härtel C. Immature anti-inflammatory response in neonates. Clin Exp Immunol (2004) 135(1):130–6. doi: 10.1111/j.1365-2249.2004.02313.x PMC1808915 14678274

[10] Wang X Mou W Qi Z Chen X Zhang H Jiao H. Neonates are armed with deviated immune cell proportion and cytokine reduction but higher T cell proliferation potentiality. Acta Biochim Biophys Sin (Shanghai) (2018) 50(9):934–7. doi: 10.1093/abbs/gmy079 30052714

[11] Li L Lee HH Bell JJ Gregg RK Ellis JS Gessner A. Il-4 utilizes an alternative receptor to drive apoptosis of Th1 cells and skews neonatal immunity toward Th2. Immunity (2004) 20(4):429–40. doi: 10.1016/s1074-7613(04)00072-x 15084272

[12] Devergne O Hummel M Koeppen H Le Beau MM Nathanson EC Kieff E. A novel interleukin-12 P40-related protein induced by latent Epstein-Barr virus infection in b lymphocytes. J Virol (1996) 70(2):1143–53. doi: 10.1128/jvi.70.2.1143-1153.1996 PMC189923 8551575

[13] Yoshida H Hunter CA. The immunobiology of interleukin-27. Annu Rev Immunol (2015) 33:417–43. doi: 10.1146/annurev-immunol-032414-112134 25861977

[14] Pflanz S Hibbert L Mattson J Rosales R Vaisberg E Bazan JF. Wsx-1 and glycoprotein 130 constitute a signal-transducing receptor for il-27. J Immunol (2004) 172(4):2225–31. doi: 10.4049/jimmunol.172.4.2225 14764690

[15] Hunter CA. New il-12-Family members: Il-23 and il-27, cytokines with divergent functions. Nat Rev Immunol (2005) 5(7):521–31. doi: 10.1038/nri1648 15999093

[16] Owaki T Asakawa M Fukai F Mizuguchi J Yoshimoto T. Il-27 induces Th1 differentiation *Via* P38 Mapk/T-bet- and intercellular adhesion molecule-1/Lfa-1/Erk1/2-Dependent pathways. J Immunol (2006) 177(11):7579–87. doi: 10.4049/jimmunol.177.11.7579 17114427

[17] Simonsen KA Anderson-Berry AL Delair SF Davies HD. Early-onset neonatal sepsis. Clin Microbiol Rev (2014) 27(1):21–47. doi: 10.1128/cmr.00031-13 24396135 PMC3910904

[18] Hartman ME Linde-Zwirble WT Angus DC Watson RS. Trends in the epidemiology of pediatric severe sepsis*. Pediatr Crit Care Med (2013) 14(7):686–93. doi: 10.1097/PCC.0b013e3182917fad 23897242

[19] Chu SM Hsu JF Lee CW Lien R Huang HR Chiang MC. Neurological complications after neonatal bacteremia: The clinical characteristics, risk factors, and outcomes. PloS One (2014) 9(11):e105294. doi: 10.1371/journal.pone.0105294 25364821 PMC4217713

[20] Seman BG Vance JK Rawson TW Witt MR Huckaby AB Povroznik JM. Elevated levels of interleukin-27 in early life compromise protective immunity in a mouse model of gram-negative neonatal sepsis. Infect Immun (2020) 88(3):e00828–19. doi: 10.1128/iai.00828-19 PMC7035946 31818960

[21] Seman BG Povroznik JM Vance JK Rawson TW. Robinson CM. A neonatal imaging model of gram-negative bacterial sepsis. J Vis Exp (2020) 162:e61609. doi: 10.3791/61609 32865536

[22] Carlson PE Jr. Carroll JA O’Dee DM Nau GJ. Modulation of virulence factors in francisella tularensis determines human macrophage responses. Microb Pathog (2007) 42(5-6):204–14. doi: 10.1016/j.micpath.2007.02.001 PMC2699611 17369012

[23] Corces MR Trevino AE Hamilton EG Greenside PG Sinnott-Armstrong NA Vesuna S. An improved atac-seq protocol reduces background and enables interrogation of frozen tissues. Nat Methods (2017) 14(10):959–62. doi: 10.1038/nmeth.4396 PMC5623106 28846090

[24] Dziadowicz SA Wang L Akhter H Aesoph D Sharma T Adjeroh DA. Bone marrow stroma-induced transcriptome and regulome signatures of multiple myeloma. Cancers (2022) 14(4):927. doi: 10.3390/cancers14040927 35205675 PMC8870223

[25] Liao Y Smyth GK Shi W. The subread aligner: Fast, accurate and scalable read mapping by seed-and-Vote. Nucleic Acids Res (2013) 41(10):e108. doi: 10.1093/nar/gkt214 23558742 PMC3664803

[26] Liao Y Smyth GK Shi W. Featurecounts: An efficient general purpose program for assigning sequence reads to genomic features. Bioinformatics (2014) 30(7):923–30. doi: 10.1093/bioinformatics/btt656 24227677

[27] Mortazavi A Williams BA McCue K Schaeffer L Wold B. Mapping and quantifying mammalian transcriptomes by rna-seq. Nat Methods (2008) 5(7):621–8. doi: 10.1038/nmeth.1226 PMC13303166 18516045

[28] Robinson MD McCarthy DJ Smyth GK. Edger: A bioconductor package for differential expression analysis of digital gene expression data. Bioinformatics (2010) 26(1):139–40. doi: 10.1093/bioinformatics/btp616 PMC2796818 19910308

[29] Liberzon A Birger C Thorvaldsdóttir H Ghandi M Mesirov JP Tamayo P. The molecular signatures database (Msigdb) hallmark gene set collection. Cell Syst (2015) 1(6):417–25. doi: 10.1016/j.cels.2015.12.004 PMC4707969 26771021

[30] Subramanian A Tamayo P Mootha VK Mukherjee S Ebert BL Gillette MA. Gene set enrichment analysis: A knowledge-based approach for interpreting genome-wide expression profiles. Proc Natl Acad Sci U.S.A. (2005) 102(43):15545–50. doi: 10.1073/pnas.0506580102 PMC1239896 16199517

[31] Huang da W Sherman BT Lempicki RA. Systematic and integrative analysis of Large gene lists using David bioinformatics resources. Nat Protoc (2009) 4(1):44–57. doi: 10.1038/nprot.2008.211 19131956

[32] Supek F Bošnjak M Škunca N Šmuc T. Revigo summarizes and visualizes long lists of gene ontology terms. PloS One (2011) 6(7):e21800. doi: 10.1371/journal.pone.0021800 21789182 PMC3138752

[33] Langmead B Trapnell C Pop M Salzberg SL. Ultrafast and memory-efficient alignment of short DNA sequences to the human genome. Genome Biol (2009) 10(3):R25. doi: 10.1186/gb-2009-10-3-r25 19261174 PMC2690996

[34] Thorvaldsdóttir H Robinson JT Mesirov JP. Integrative genomics viewer (Igv): High-performance genomics data visualization and exploration. Brief Bioinform (2013) 14(2):178–92. doi: 10.1093/bib/bbs017 PMC3603213 22517427

[35] Zhang Y Liu T Meyer CA Eeckhoute J Johnson DS Bernstein BE. Model-based analysis of chip-seq (Macs). Genome Biol (2008) 9(9):R137. doi: 10.1186/gb-2008-9-9-r137 18798982 PMC2592715

[36] McLean CY Bristor D Hiller M Clarke SL Schaar BT Lowe CB. Great improves functional interpretation of cis-regulatory regions. Nat Biotechnol (2010) 28(5):495–501. doi: 10.1038/nbt.1630 20436461 PMC4840234

[37] Jassal B Matthews L Viteri G Gong C Lorente P Fabregat A. Reactome pathway knowledgebase. Nucleic Acids Res (2020) 48(D1):D498–d503. doi: 10.1093/nar/gkz1031 31691815 PMC7145712

[38] Li Y Zhang P Wang C Han C Meng J Liu X. Immune responsive gene 1 (Irg1) promotes endotoxin tolerance by increasing A20 expression in macrophages through reactive oxygen species. J Biol Chem (2013) 288(23):16225–34. doi: 10.1074/jbc.M113.454538 PMC3675562 23609450

[39] Michelucci A Cordes T Ghelfi J Pailot A Reiling N Goldmann O. Immune-responsive gene 1 protein links metabolism to immunity by catalyzing itaconic acid production. Proc Natl Acad Sci USA (2013) 110(19):7820–5. doi: 10.1073/pnas.1218599110 PMC3651434 23610393

[40] Wullaert A Verstrepen L Van Huffel S Adib-Conquy M Cornelis S Kreike M. Lind/Abin-3 is a novel lipopolysaccharide-inducible inhibitor of nf-kappab activation. J Biol Chem (2007) 282(1):81–90. doi: 10.1074/jbc.M607481200 17088249

[41] Yanagisawa H Davis EC. Unraveling the mechanism of elastic fiber assembly: The roles of short fibulins. Int J Biochem Cell Biol (2010) 42(7):1084–93. doi: 10.1016/j.biocel.2010.03.009 PMC2880191 20236620

[42] Li J Wang X Ackerman WET Batty AJ Kirk SG White WM. Dysregulation of lipid metabolism in mkp-1 deficient mice during gram-negative sepsis. Int J Mol Sci (2018) 19(12):3904. doi: 10.3390/ijms19123904 30563203 PMC6321205

[43] Rhoads JP Major AS. How oxidized low-density lipoprotein activates inflammatory responses. Crit Rev Immunol (2018) 38(4):333–42. doi: 10.1615/CritRevImmunol.2018026483 PMC6527110 30806246

[44] Go GW Mani A. Low-density lipoprotein receptor (Ldlr) family orchestrates cholesterol homeostasis. Yale J Biol Med (2012) 85(1):19–28. 22461740 PMC3313535

[45] DeGregori J Leone G Miron A Jakoi L Nevins JR. Distinct roles for E2f proteins in cell growth control and apoptosis. Proc Natl Acad Sci U.S.A. (1997) 94(14):7245–50. doi: 10.1073/pnas.94.14.7245 PMC23805 9207076

[46] Pearl JE Khader SA Solache A Gilmartin L Ghilardi N deSauvage F. Il-27 signaling compromises control of bacterial growth in mycobacteria-infected mice. J Immunol (2004) 173(12):7490–6. doi: 10.4049/jimmunol.173.12.7490 15585875

[47] Hölscher C Hölscher A Rückerl D Yoshimoto T Yoshida H Mak T. The il-27 receptor chain wsx-1 differentially regulates antibacterial immunity and survival during experimental tuberculosis. J Immunol (2005) 174(6):3534–44. doi: 10.4049/jimmunol.174.6.3534 15749890

[48] Robinson CM Nau GJ. Interleukin-12 and interleukin-27 regulate macrophage control of mycobacterium tuberculosis. J Infect Dis (2008) 198(3):359–66. doi: 10.1086/589774 PMC2761687 18557702

[49] Robinson CM Jung JY Nau GJ. Interferon-Γ, tumor necrosis factor, and interleukin-18 cooperate to control growth of mycobacterium tuberculosis in human macrophages. Cytokine (2012) 60(1):233–41. doi: 10.1016/j.cyto.2012.06.012 PMC3429699 22749533

[50] Jung JY Robinson CM. Interleukin-27 inhibits phagosomal acidification by blocking vacuolar atpases. Cytokine (2013) 62(2):202–5. doi: 10.1016/j.cyto.2013.03.010 PMC3760007 23557795

[51] Jung JY Robinson CM. Il-12 and il-27 regulate the phagolysosomal pathway in mycobacteria-infected human macrophages. Cell Commun Signal (2014) 12:16. doi: 10.1186/1478-811x-12-16 24618498 PMC4007735

[52] Roth J Vogl T Sorg C Sunderkötter C. Phagocyte-specific S100 proteins: A novel group of proinflammatory molecules. Trends Immunol (2003) 24(4):155–8. doi: 10.1016/s1471-4906(03)00062-0 12697438

[53] Vogl T Tenbrock K Ludwig S Leukert N Ehrhardt C van Zoelen MA. Mrp8 and Mrp14 are endogenous activators of toll-like receptor 4, promoting lethal, endotoxin-induced shock. Nat Med (2007) 13(9):1042–9. doi: 10.1038/nm1638 17767165

[54] Ding Z Du F Averitt VR Jakobsson G Rönnow CF Rahman M. Targeting S100a9 reduces neutrophil recruitment, inflammation and lung damage in abdominal sepsis. Int J Mol Sci (2021) 22(23):12923. doi: 10.3390/ijms222312923 34884728 PMC8658007

[55] Hao J Hu Y Li Y Zhou Q Lv X. Involvement of jnk signaling in Il4-induced M2 macrophage polarization. Exp Cell Res (2017) 357(2):155–62. doi: 10.1016/j.yexcr.2017.05.010 28501460

[56] Villarino A Hibbert L Lieberman L Wilson E Mak T Yoshida H. The il-27r (Wsx-1) is required to suppress T cell hyperactivity during infection. Immunity (2003) 19(5):645–55. doi: 10.1016/s1074-7613(03)00300-5 14614852

[57] Hamano S Himeno K Miyazaki Y Ishii K Yamanaka A Takeda A. Wsx-1 is required for resistance to trypanosoma cruzi infection by regulation of proinflammatory cytokine production. Immunity (2003) 19(5):657–67. doi: 10.1016/s1074-7613(03)00298-x 14614853

[58] Artis D Villarino A Silverman M He W Thornton EM Mu S. The il-27 receptor (Wsx-1) is an inhibitor of innate and adaptive elements of type 2 immunity. J Immunol (2004) 173(9):5626–34. doi: 10.4049/jimmunol.173.9.5626 15494513

[59] Rosas LE Satoskar AA Roth KM Keiser TL Barbi J Hunter C. Interleukin-27r (Wsx-1/T-Cell cytokine receptor) gene-deficient mice display enhanced resistance to leishmania donovani infection but develop severe liver immunopathology. Am J Pathol (2006) 168(1):158–69. doi: 10.2353/ajpath.2006.050013 PMC1592683 16400019

[60] Wirtz S Tubbe I Galle PR Schild HJ Birkenbach M Blumberg RS. Protection from lethal septic peritonitis by neutralizing the biological function of interleukin 27. J Exp Med (2006) 203(8):1875–81. doi: 10.1084/jem.20060471 PMC2118378 16880260

[61] Patel VP Kreider BL Li Y Li H Leung K Salcedo T. Molecular and functional characterization of two novel human c-c chemokines as inhibitors of two distinct classes of myeloid progenitors. J Exp Med (1997) 185(7):1163–72. doi: 10.1084/jem.185.7.1163 PMC2196270 9104803

[62] Baorto DM Gao Z Malaviya R Dustin ML van der Merwe A Lublin DM. Survival of fimh-expressing enterobacteria in macrophages relies on glycolipid traffic. Nature (1997) 389(6651):636–9. doi: 10.1038/39376 9335508

[63] McArdel SL Terhorst C Sharpe AH. Roles of Cd48 in regulating immunity and tolerance. Clin Immunol (2016) 164:10–20. doi: 10.1016/j.clim.2016.01.008 26794910 PMC4860950

[64] Kirschning CJ Schumann RR. Tlr2: Cellular sensor for microbial and endogenous molecular patterns. Curr Top Microbiol Immunol (2002) 270:121–44. doi: 10.1007/978-3-642-59430-4_8 12467248

[65] Buwitt-Beckmann U Heine H Wiesmüller KH Jung G Brock R Akira S. Tlr1- and Tlr6-independent recognition of bacterial lipopeptides. J Biol Chem (2006) 281(14):9049–57. doi: 10.1074/jbc.M512525200 16455646

[66] Feng D Yu J Bao L Fan D Zhang B. Inhibiting Rgs1 attenuates secondary inflammation response and tissue degradation *via* the Tlr/Trif/Nf-κb pathway in macrophage post spinal cord injury. Neurosci Lett (2022) 768:136374. doi: 10.1016/j.neulet.2021.136374 34852285

[67] Cheung H Chen NJ Cao Z Ono N Ohashi PS Yeh WC. Accessory protein-like is essential for il-18-Mediated signaling. J Immunol (2005) 174(9):5351–7. doi: 10.4049/jimmunol.174.9.5351 15843532

[68] Bermudes ACG de Carvalho WB Zamberlan P Muramoto G Maranhão RC Delgado AF. Changes in lipid metabolism in pediatric patients with severe sepsis and septic shock. Nutrition (2018) 47:104–9. doi: 10.1016/j.nut.2017.09.015 29429528

