# Supplementary material for: Interleukin-27-dependent transcriptome signatures during neonatal sepsis
Source: Front Immunol. 2023 Feb 20;14:1124140. doi: 10.3389/fimmu.2023.1124140 (PMC9986606; doi:10.3389/fimmu.2023.1124140)

Supplementary Figure 1. Biochemical analysis of IL-27Rα in neonatal splenocytes. Flow cytometric analysis of uninfected WT **(A)** and IL-27Rα-deficient **(B)** neonatal murine splenocytes from post-natal day 7-8 pups revealed only background signal for IL-27Rα in splenocytes from KO pups **(D)** relative to WT counterparts **(C)**. **(E)** Immunoblot analysis of IL-27Rα demonstrated an absence of receptor in adult and neonatal KO splenic lysates compared to WT mice. Lane 1: 50 µg adult WT lysate; Lane 2: 50 µg adult KO lysate; Lane 3: 20 µg neonate WT lysate; Lane 4: 20 µg neonate KO lysate. WT-A: WT-Adult; KO-A: KO-Adult; WT-N: WT-Neonate; KO-N: KO-Neonate.

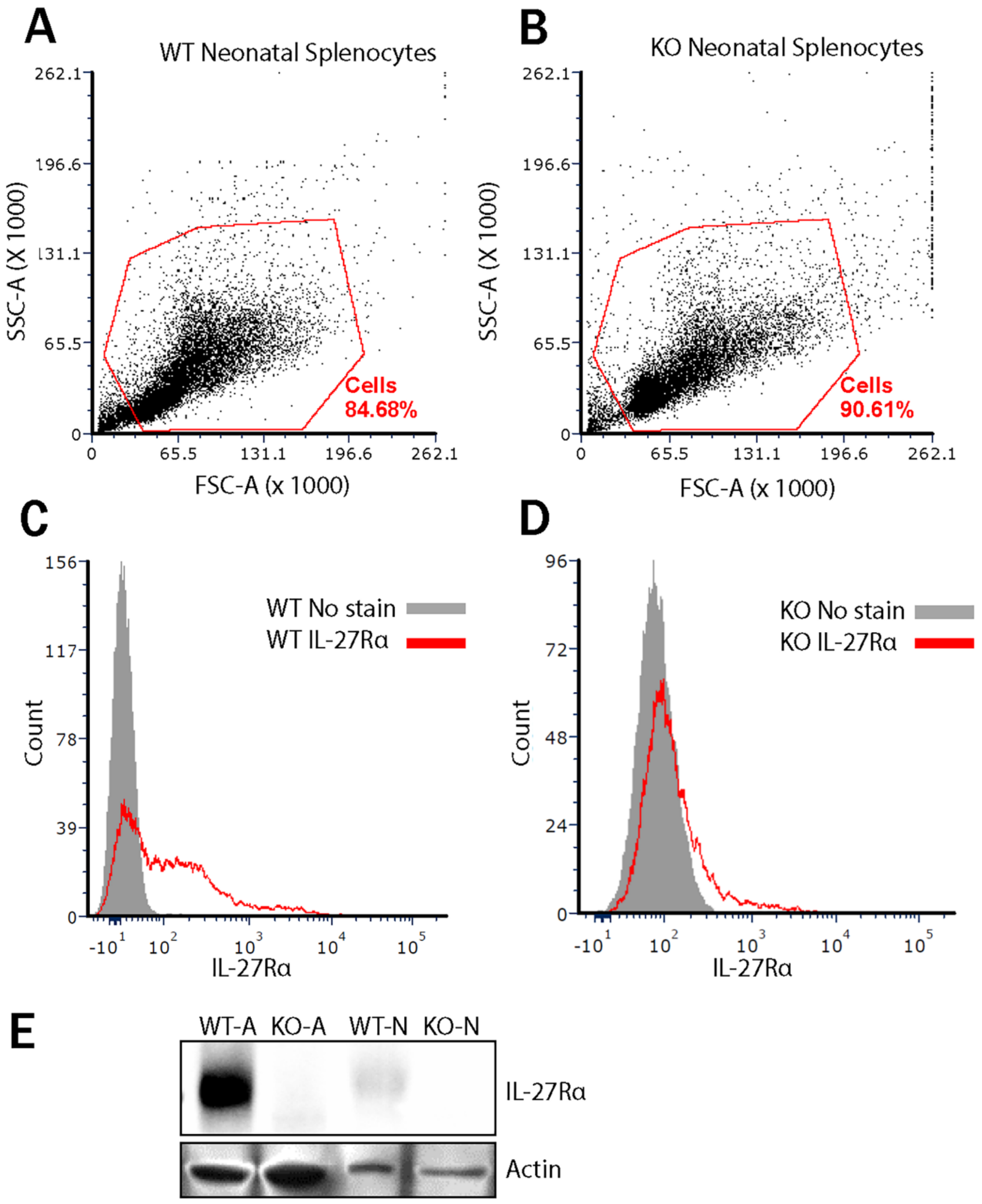

Supplementary Figure 2. Functional analysis of genes downregulated by *E. coli* infection in the spleen tissue. **(A)** Top 10 gene sets for Reactome pathways that are downregulated in the samples treated with *E. coli* as predicted from GSEA (FDR q < 0.01). NES: normalized enrichment score. **(B)** GSEA analysis for expressed genes ranked by fold change of expression (*E. coli*/Ctrl) against the Reactome pathway "Activation of the pre replicative complex", with top 10 down-regulated genes shown at the bottom. **(C)** Top 10 gene sets for gene ontologies in biological processes that are downregulated in samples treated with *E. coli* as predicted from GSEA (FDR q < 0.01). **(D)** GSEA analysis for expressed genes ranked by fold change of expression (*E. coli*/Ctrl) against the biological process "DNA replication", with top 10 down-regulated genes shown at the bottom.

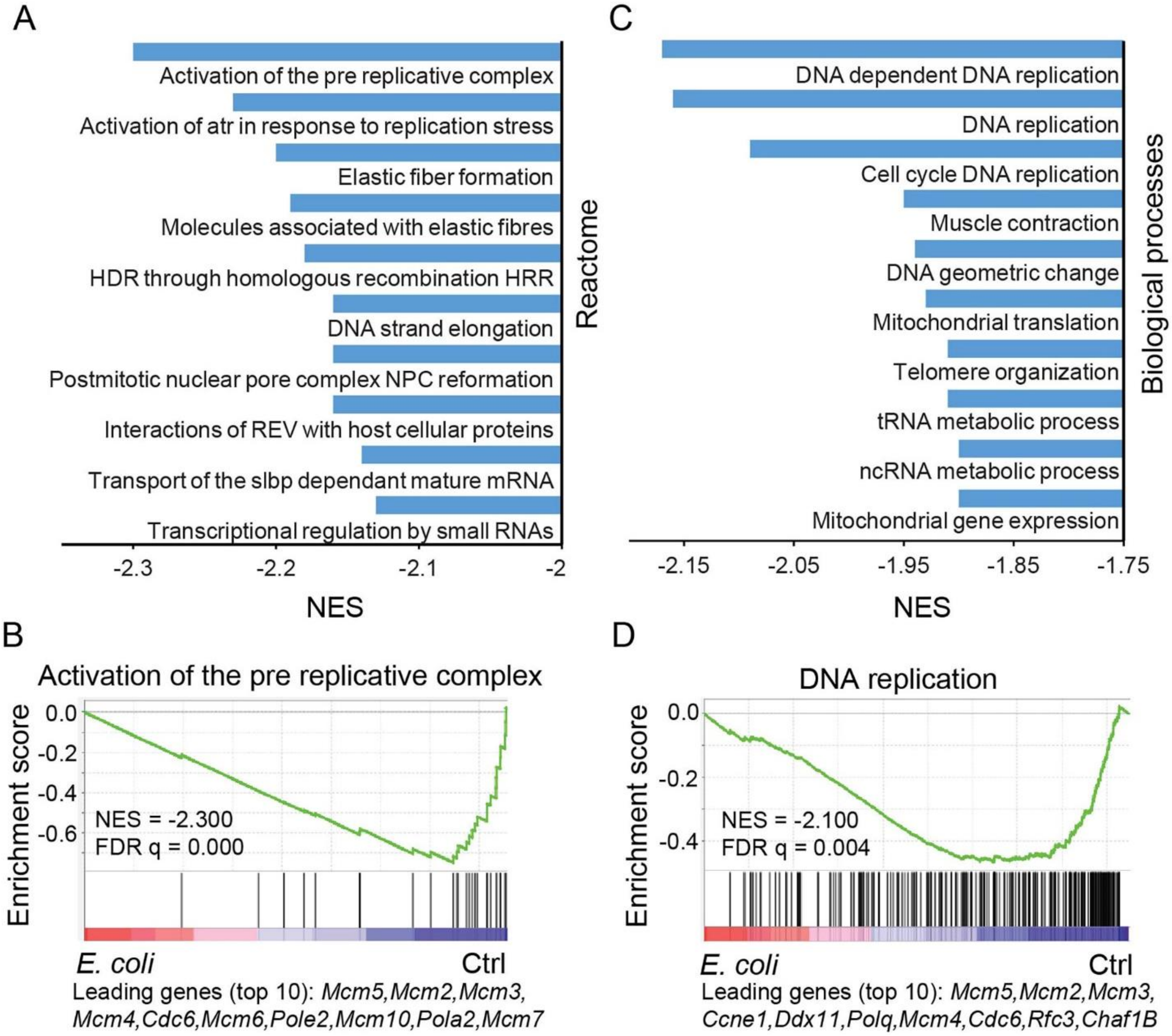

Supplementary Figure 3. Transcriptional response to *E. coli* infection in the liver (a re-analysis of RNA-Seq expression data from GEO GSE122741). **(A)** Volcano plot for genes differentially expressed between *E. coli* treated and control liver samples. Blue +: genes upregulated after *E. coli* infection; red -: genes down-regulated after *E. coli* infection. Blue smooth heat map: all expressed genes. **(B)** Top 10 gene sets on gene ontologies in biological processes that are upregulated in the liver samples treated with *E. coli* as predicted from GSEA (FDR q < 0.01). NES: normalized enrichment score. **(C)** Top 10 gene sets on gene ontologies in biological processes that are downregulated in the liver samples treated with *E. coli* as predicted from GSEA (FDR q < 0.01). **(D)** Venn diagrams for the numbers of genes commonly or specifically up-regulated or down-regulated by *E. coli* infection between liver and spleen. **(E)** Gene ontology analysis on biological processes (BP3) for genes commonly upregulated in spleen and liver by *E. coli*, with results being summarized as a scatterplot of clustering of enriched terms based on semantic similarity. Circle size represents $-\log_{10}$(P-value) corresponding to the significance of enrichment.

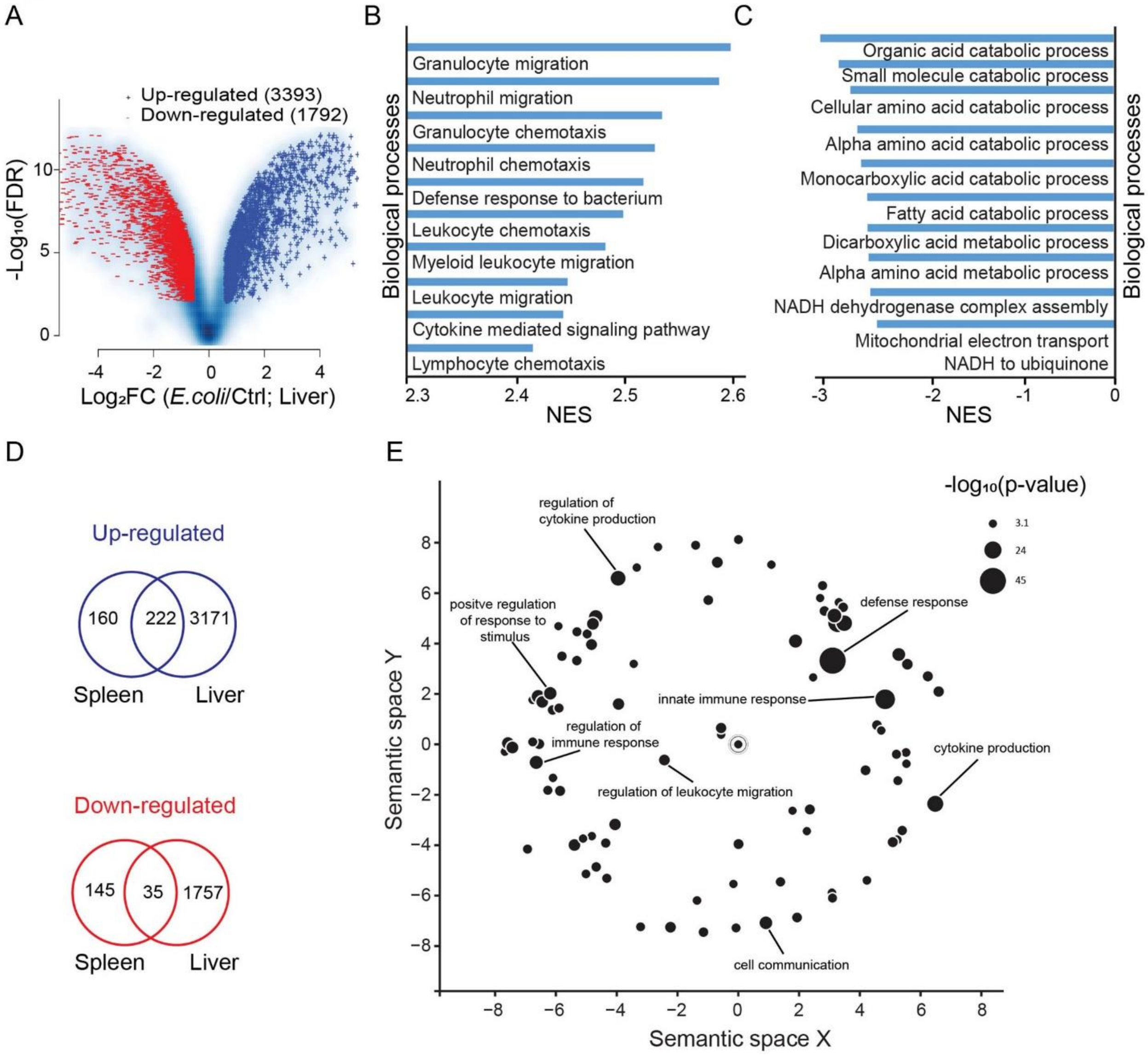

Supplementary Figure 4. Up-regulated pathways and biological process in IL27Rα KO cells as compared to WT cells post *E. coli* infection. **(A)** Top 10 gene sets on Reactome pathways that are up-regulated in the samples with IL27Rα-KO post *E. coli* infection (FDR q < 0.01). NES: normalized enrichment score. **(B)** Top 10 gene sets on gene ontologies in biological processes that are upregulated in samples with IL27Rα-KO post *E. coli* infection (FDR q < 0.01). **(C)** Gene ontology analysis on biological processes (BP3) for genes that failed to upregulate in expression during *E. coli* infection in the absence of IL27Rα, with results summarized as a scatterplot of clustering of enriched terms based on semantic similarity. Circle size represents $-\log_{10}$(P-value) corresponding to the significance of enrichment.

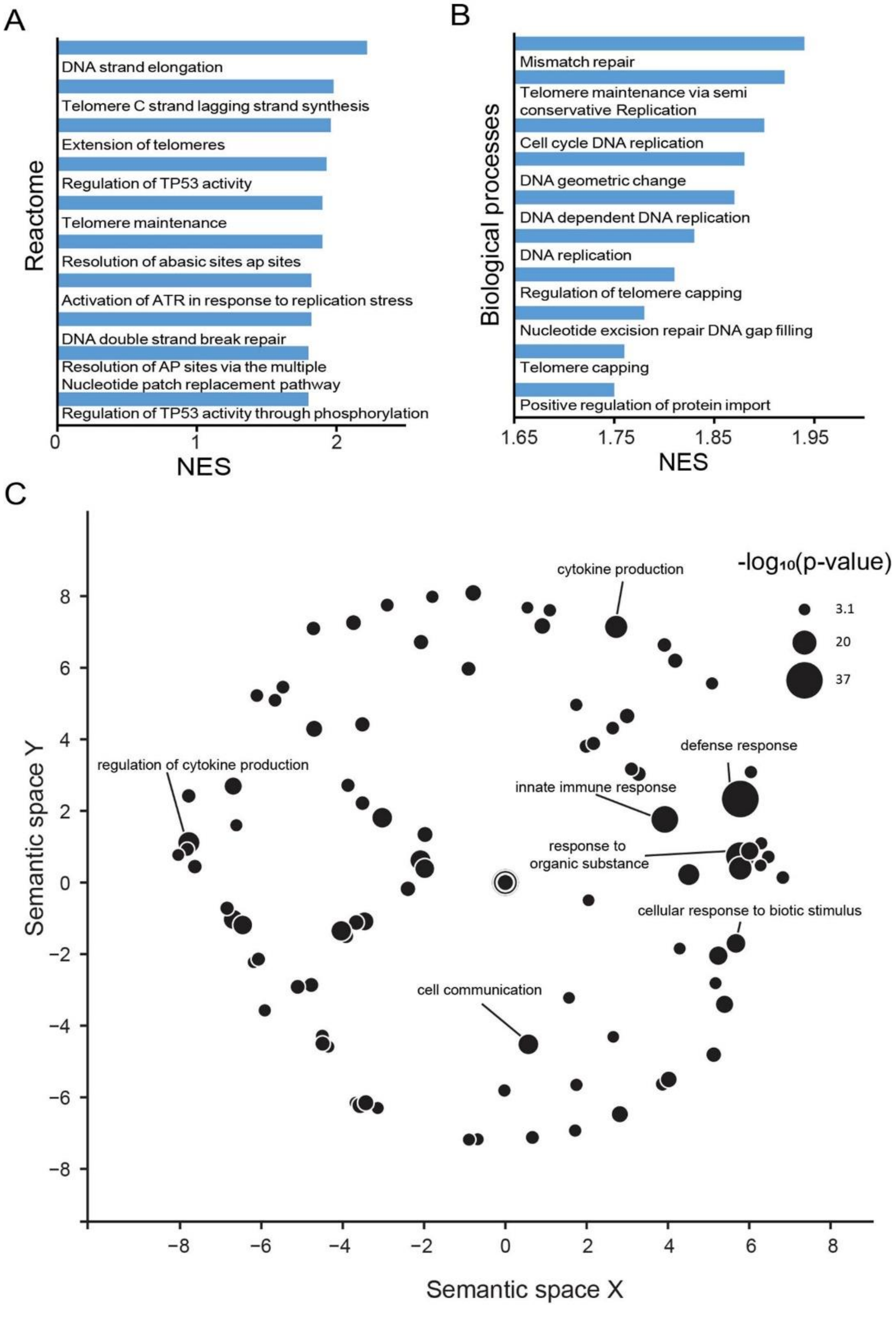

Supplementary Figure 5. Compromised regulome response to *E. coli* in macrophage-enriched cells deficient in IL-27Rα. (**A**) Genome browser image for the distribution of ATAC-seq read across genomic regions enclosing *s100a9* (left panel) and *mrc1* (right panel) for WT macrophages from mice with and without *E. coli* infection (Ctrl). Genomic regions changing in chromatin accessibility in response to *E. coli* are shadowed in yellow. Expression values (RPKM in $\log_2$) also indicated. (**B**) GSEA analysis for expressed genes ranked by the fold change of expression (*E. coli*/Ctrl in macrophages) from high to low on the red-to-blue spectrum against genes associated with multiple genomic regions showing increase (left panel) or decrease (right panel) in chromatin accessibility in response to *E. coli* infection, with both changes measured in WT macrophages. (**C**) Boxplot of *E. coli*-induced changes in chromatin accessibility in IL-27Rα-deficient macrophages (KO) and in WT macrophages for open chromatin regions sorted based on their changes in accessibility in response to *E. coli* in WT macrophages. *P*-value by paired *t*-test.

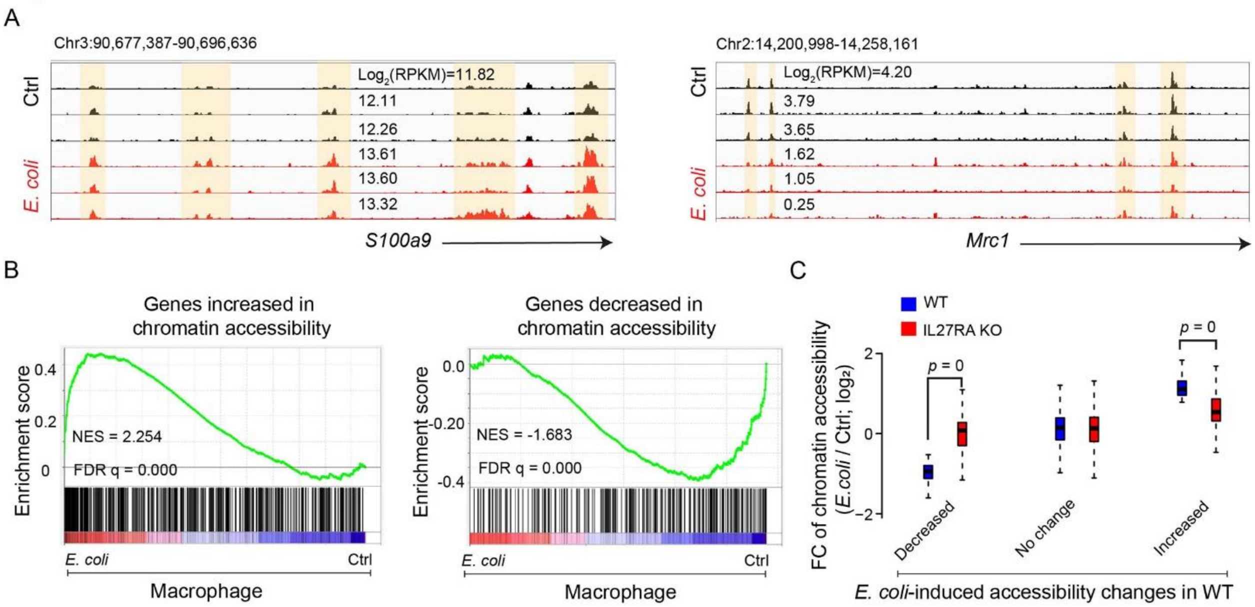

Supplement: Supplementary file 2 [file DataSheet_1.pdf]
